# Which is the best for the warfarin monitoring: Following up by fixed or variable physician?

**DOI:** 10.14744/nci.2021.06981

**Published:** 2022-03-10

**Authors:** Lale Dinc Asarcikli, Habibe Kafes, Taner Sen, Esra Gucuk Ipek, Osman Beton, Ahmet Temizhan, Mehmet Birhan Yilmaz

**Affiliations:** 1 Department of Cardiology, Dr. Siyami Ersek Thoracic Surgery Center, Istanbul, Turkey; 2 Department of Cardiology, University of Health Sciences, Ankara City Hospital, Ankara, Turkey; 3 Department of Cardiology, Dumlupinar University Evliya Celebi Training and Research Hospital, Kutahya, Turkey; 4 Department of Cardiology, Polatli State Hospital, Ankara, Turkey; 5 Department of Cardiology, Cumhuriyet University Faculty of Medicine, Sivas, Turkey

**Keywords:** Anticoagulation, embolism, hemorrhage, international normalized ratio, warfarin

## Abstract

**Objective:**

Warfarin therapy has some difficulties in terms of close monitoring and dosage. This study aims to evaluate the effect of same-fixed versus different-variable physician-based monitoring of warfarin therapy on treatment quality and clinical end-points.

**Methods:**

A total of 625 consecutive patients requiring warfarin treatment were enrolled at seven centers. INR values of the patients measured at each visit and registered to hospital database were recorded. Time in therapeutic range (TTR) was calculated using linear interpolation method (Rosendaal’s method). A TTR value of ≥65% was considered as effective warfarin treatment. If a patient was evaluated by the same-fixed physician at each INR visit, was categorized into the same-physician (SP) group. In contrast, if a patient was evaluated by different-variable physicians at each INR visit, was categorized into variable physician (VP) group. Enrolled patients were followed up for bleeding and embolic events.

**Results:**

One hundred and fifty-six patients (24.9%) were followed by SP group, 469 (75.1%) patients were followed by VP group. Median TTR value of the VP group was lower than that of SP group (56.2% vs. 65.1%, respectively, p=0.009). During median 25.5 months (9–36) of follow-up, minor bleeding, major bleeding and cerebral embolic event rates were higher in VP group compared to SP group (p<0.001, p=0.023, p<0.001, respectively). In multivariate analysis, INR monitoring by VP group was found to be an independent predictor of increased risk of bleeding events (OR 2.55, 95% CI 1.64–3.96, p<0.001) and embolism (OR 3.42, 95% CI 1.66–7.04, p=0.001).

**Conclusion:**

INR monitoring by same physician was associated with better TTR and lower rates of adverse events during follow-up. Hence, it is worth encouraging an SP-based outpatient follow-up system at least for where warfarin therapy is the only choice.

Warfarin is the most frequently used medication to prevent thromboembolic events in patients with atrial fibrillation (AF), prosthetic heart valves and venous thrombosis [1]. Although the clinical efficacy of warfarin on preventing the thromboembolic events has already been well established, many patients are left untreated due to well-known drawbacks of warfarin therapy [2]. The narrow therapeutic range, interactions with other drugs and nutritional vitamin K, pharmacogenetic properties, the necessity of frequent monitoring and serious complications are counted among the limiting factors for warfarin usage [3]. The quality of treatment can be measured both by the frequency of complications and by measuring the proportion of daily values within a strict range of target international normalized ratio (INR) namely, “Time in Therapeutic Range” (TTR) [4]. An optimum TTR correlates with a low risk of warfarin-related complications [5]. A 10% difference in TTR leads to increased risk of 1.29 for mortality [6].

Warfarin monitoring requires experienced staff and a good physician/health personnel-patient relationship for a better treatment adherence. Studies evaluating the quality of warfarin treatment found that patient related factors such as patient knowledge and compliance level are associated with the success of warfarin treatment [7]. In many countries, anticoagulation centers or home-based monitoring systems are not available. In addition, in some centers, warfarin monitoring is carried out by irregular visits and by various primary physicians. It is unclear whether these factors are associated with the failure of achieving optimum TTR or with warfarin-related adverse events. In the present study, we aimed to evaluate and compare the quality of warfarin monitoring by same-fixed physician versus by different-variable physician-based through measuring the quality of TTR as well as clinical outcomes during follow-up in outpatient clinics.

## Materials and Methods

We conducted an observational multicenter prospective cohort study. Consecutive patients admitting to cardiology out-patient clinics at seven high-volume health care centers between March 2012 and March 2015 with age ≥18 years who were on warfarin treatment for at least 6 months with indications of valvular/non-valvular AF, prosthetic heart valve, deep vein thrombosis, and pulmonary embolism. Patients with inadequate regular follow-up with more than 60 days interval and lack of at least 4 registered INR value, malignancy or hospitalization due to any cause in the previous 6 months, interruption of warfarin therapy for any cause, presence of active infection, hepatitis or chronic liver failure and patients who couldn’t come to follow up visits regularly or patients with a life expectancy <6 months were excluded from the study. Patients who were not followed up by a single center were excluded. At least 4 INR values of the patients were obtained retrospectively from hospital database at index visit. At each visit, INR values of the patients and dates (day/month/year) of INR measurement were recorded and target INR ranges for each patient were entered. The TTR was measured using “Rosendaal linear interpolation” method [4]. Effective TTR was accepted as ≥65% in TTR in accordance with the recommendations of guidelines [8].

**Highlight key points**

• Warfarin monitoring by fixed physician is an effective, feasible and safe method.
• Better TTR values and lesser complications with same-fixed physician.
• Higher confidence level about warfarin monitoring can be achieved in patients followed by same-fixed physician.

Physicians participating in the present study were only cardiologists. Three centers had multiple cardiologists with weekly or monthly shifts in their outpatient clinics. Hence, patients were exposed to different physicians during outpatient visits for warfarin monitoring. These patients were categorized into “variable-physician (VP) group.” The remaining four centers provided a stable physician (cardiologist) at their outpatient clinic for warfarin monitoring. Hence, each patient was exposed to same-fixed physician during different visits. These patients were categorized into “same-physician (SP) group.”

The study was conducted in compliance with the principles included the Declaration of Helsinki and approved by the local ethics committee (Registration number: 15-EPKK-619-6689). All patients signed an informed consent.

### Definitions

Bleeding events were classified according to BARC bleeding classification [9]. Type 1 and 2 bleedings were referred to as “minor bleeding” whereas type 3–5 bleedings were referred to as “major bleeding. Ischemic complications were classified as cerebral embolism (acute ischemic stroke or transient ischemic attack) and peripheral embolism (acute mesenteric ischemia, acute limb ischemia, acute myocardial infarction), caused by arterial thromboembolism that leads to organ-specific ischemia [10]. Target INR values were assessed according to current guidelines [3, 11, 12]. Congestive heart failure, Hypertension, Age ≥75 years, Diabetes mellitus, Prior Stroke or TIA or Thromboembolism, Vascular disease, Age 65–74 years, Female Sex (CHA_2_DS_2_-VASc score) was used for the determination of stroke risk for warfarin indication in patients with AF [13]. HAS-BLED and anemia, severe renal disease, age ≥75 years, any prior hemorrhage, hypertension history (ATRIA) bleeding scores were used for the evaluation of the bleeding risk and HAS-BLED ≥3 was considered high risk for major bleeding [14].

Duration of warfarin usage and frequency of INR measurements was recorded according to patients’ self-report. There is no standard scale for measuring the level of confidence (patients’ trust in the effectiveness of warfarin treatment) about warfarin monitoring. We used a straightforward numerical Likert scale of 0–10 in which 0 represents no confidence and 10 represents complete confidence. Level of confidence score was categorized into 5 levels: No (0–2), little (3–4), moderate (5–6), moderate to high (7–8), and high (9–10).

Estimated glomerular filtration rate (eGFR) which was calculated by Modification of Diet in Renal Disease formula, eGFR with <30 ml/min/1.73m^2^ was defined as severe chronic renal disease and eGFR with <15 ml/min/1.73m^2^ or requiring dialysis was defined as end-stage chronic renal disease [15].

### Data Collection

Demographics including educational status and monthly income, clinical history, cardiovascular risk factors, concomitant medications, the frequency of INR controls per year, history of bleeding, or thromboembolic complications were recorded at the index visit. Patients were asked about the concomitant anti-platelet drugs (acetylsalicylic acid, clopidogrel, dipyridamole, etc.) or non-steroidal anti-inflammatory drug usage, usage of herbal supplements and changes in dietary habits in each visit. INR measurement was done in each center within their own laboratories. Echocardiographic measurements were performed according to guidelines [16]. Patients were followed up for median 25.5 months (9–36) and questioned about events at each visit or through phone calls by a study coordinator who was appointed for the recording of outcome data. After collecting outcome data, an experienced investigator, blinded to the study plan, adjudicated the data according to definitions.

### Statistical Analysis

Statistical analyses were performed using SPSS version 19.0 (IBM SPSS Statistics, IBM Corporation, Chicago, IL, USA). Categorical variables were presented as percentages and numbers; continuous variables were presented as mean±standard deviation or median (minimum–maximum). Test of normality (Kolmogorov-Smirnov statistic, with a Lilliefors significance level) was performed for all continuous variables. Comparison between groups of patients was made using a χ^2^ test, independent samples t-test, and Mann-Whitney U test, as appropriate. Univariate logistic regression analysis was used to quantify the association of variables with the occurrence of bleeding and embolism. Variables included in the multivariate analysis were those reaching a significance level p≤0.1 on univariate analysis. Results of the multivariate analysis were reported as odds ratio (OR) with corresponding 95% confidence interval and P value. A p≤0.05 was considered significant.

## Results

**Figure 1.**
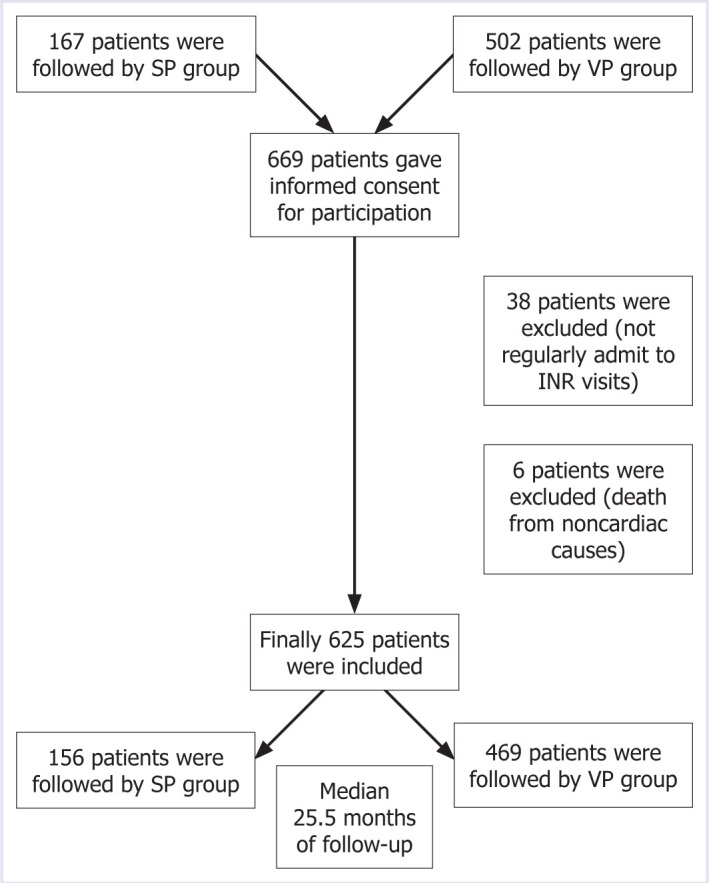
Flow chart of study population selection. SP: Same/fixed-physician; VP: Different/variable-physician.

The study population consisted of 625 consecutive patients who met the inclusion criteria as shown in Figure 1. Warfarin monitoring of 156 (24.9%) patients were done by SP group and of 469 (75.1%) patients were done by VP group. Patients’ characteristics were presented in Table 1. There were 224 (91.4%) males in VP group, whereas 45 males (28.8%) in the SP group (p<0.001). There were no statistically significant differences between groups in terms of age and distribution of comorbidities such as coronary artery disease, systolic heart failure, severe renal disease, hypertension, diabetes, hyperlipidemia, obesity, alcohol consumption, smoking status, presence of active gastric ulcer, and usage of antiplatelet and lipid-lowering drugs. The median HASBLED score was lower in VP group than in SP group (1 vs. 2, p=0.002). Patients with HASBLED score ≥3 were lower in VP group than in SP group (13.6% vs. 23.1%, p=0.006). The median ATRIA bleeding score was statistically lower in VP group than in SP group (1 vs. 2, p=0.005). The median CHA_2_DS_2_-VASc score was similar between the two groups. There was no statistically significant difference concerning warfarin indications between the two groups. The median total weekly warfarin dose was similar between groups. The median duration of warfarin use was longer and the median number of annual INR measurements was higher in the VP group compared to SP group (p=0.002 and p=0.007, respectively). The median TTR was significantly lower in VP group compared to SP group (56.2 vs. 65.1, p=0.009). Effective TTR (TTR ≥65%) tended to be less frequently achieved in the VP group compared to the SP group (39.7% vs. 51.2%, p=0.162). Among patients’ socioeconomic characteristics, high educational level and high monthly income level were more frequently noted in the VP group compared to SP group (17.1% vs. 8.3%, p<0.008 and 12.4% vs. 2.6%, p=0.001, respectively). Moderate to high and high level of confidence about warfarin monitoring were less frequently noted in the VP group compared to SP group (19.6% vs. 32.7%, p=0.001 and 11.5% vs. 20.5%, p=0.006, respectively).

**Table 1.** Characteristics of study population according to group of monitoring physician

Variables	Stable physician (n=156)	Variable physician (n=469)	p
Age (year)	61.0±12.0	60.2±13.2	0.311
Male gender (%)	28.8	47.8	<0.001
High education level* (%)	8.3	17.1	0.008
Monthly income (>500 Euro) (%)	2.6	12.4	0.001
Hypertension (%)	46.8	43.1	0.417
Diabetes mellitus (%)	16.0	18.8	0.442
Obesity (%)	14.7	19.4	0.212
Hypercholesterolemia (%)	29.9	31.1	0.311
Lipid-lowering drug (%)	20.8	26.7	0.142
Smoking (%)	23.7	26.0	0.389
Alcohol consumption (%)	3.2	2.3	0.566
Coronary artery disease (%)	12.8	13.4	0.609
Active gastric ulcer (%)	7.7	7.0	0.924
Systolic heart failure (%)	19.2	21.7	0.182
Severe renal disease (%)	0.6	0.4	0.952
Antiplatelet drugs (%)	27.3	32.6	0.214
NSAID (2–3 times/week) (%)	23.7	20.0	0.178
HASBLED score	0–3	0–4	0.002
HASBLED Score ≥3 (%)	23.1	13.6	0.005
ATRIA bleeding risk score	0–7	0–8	0.006
CHA_2_DS_2_-VASc Score	0–8	0–8	0.912
CHA_2_DS_2_-VASc Score ≥2 (%)	66	65.9	0.968
Indication for warfarin use (%)			0.256
Mechanical valve prosthesis	50.0	46.9	
Valvular AF	17.9	20.0	
Non-valvular AF	28.8	25.8	
Thromboembolic events (without AF)	3.3	8.3	
Duration of warfarin use (year)	4.2 (5–25)	4.2 (5–35)	0.002
Frequency of INR measurement/year	10.1 (6–30)	12.3 (6–28)	0.007
Total warfarin dose/week (mg)	35.2 (4–80)	32.5 (3.2–140)	0.821
TTR	65.1 (0–100)	56.2 (0–100)	0.009
TTR >65% (%)	51.2	39.7	0.162
Level of confidence about warfarin monitoring (%)			
None	10.3	11.7	0.668
Little	8.3	31.7	<0.001
Moderate	28.2	23.9	0.338
Moderate to high	32.7	19.6	0.001
High	20.5	11.5	0.006
Awareness of target INR value (%)	25.6	19.8	0.116

*: High school-college-university; NSAID: Non-steroidal anti-inflammatory drug; HASBLED: Hypertension history, abnormal liver and renal function, stroke history, bleeding history, labile INR, elderly, drugs; ATRIA bleeding score: Anemia, severe renal disease, age ≥75 years, any prior hemorrhage, hypertension history, CHA_2_DS_2_-VASc: Congestive heart failure; Hypertension: Age ≥75 years, diabetes mellitus, Prior stroke or TIA or thromboembolism, vascular disease, Age 65–74 years, Sex, AF: Atrial fibrillation; INR: International normalized ratio; TTR: Time in therapeutic range.

### Bleeding and Embolic Events

**Figure 2.**
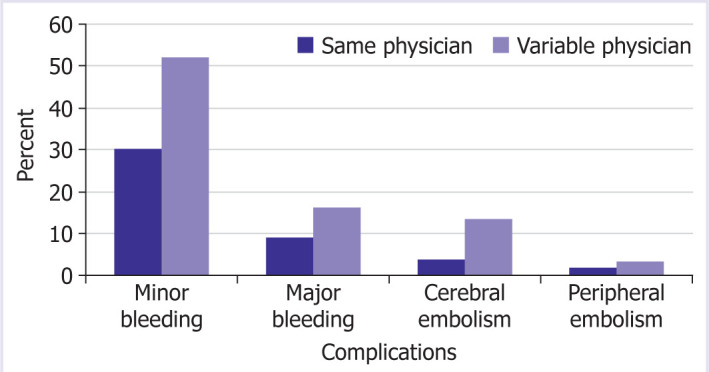
Complication rates during follow-up period according to the following physician.

Characteristics of the patients according to events during follow-up were presented in Table 2 and Figure 2. Overall, 256 minor bleeding, 90 major bleeding, 69 cerebral embolism, and 19 peripheral embolism events were adjudicated by the blinded author at the end of the follow-up period. Minor bleeding and major bleeding events were more frequently recorded in the VP group compared to SP group (47.8% vs. 20.5%, p<0.001 and 16.2% vs. 9.0%, p=0.024, respectively). Patients with bleeding complications (both minor and major bleeding) had higher HASBLED and ATRIA bleeding scores, longer duration of warfarin usage, more gastric ulcers, and more frequent in the VP group compared to patients without bleeding (Table 3). Embolic events were noted in 88 (14%) patients during follow-up. Embolic events overall were more frequent in the VP group compared to SP group (88.6% vs. 72.8%, p=0.001). Cerebral embolism was more frequent in the VP group compared to SP group (13.4% vs. 3.8%, p<0.001). Of note, median CHA_2_DS_2_-VASc score was higher in patients with embolic complications (3 vs. 2, p=0.002). Awareness of INR target was noted to be lower in patients with embolic events compared to those without embolic events (11.4% vs. 22.9%, p=0.03).

**Table 2.** Bleeding and embolic events during warfarin use

Events	Stable physician group (n=156)	Variable physician group (n=469)	p
Minor bleeding (%)	20.5	47.8	<0.001
Major bleeding (%)	9.0	16.2	0.023
Cerebral embolism (%)	3.8	13.4	<0.001
Peripheral embolism (%)	1.9	3.4	0.609

**Table 3.** Univariate and multivariate predictors of bleeding events*

Variables	Univariate			Multivariate		
	OR	95% CI	p	OR	95% CI	p
Variable physicians	2.41	1.66–3.50	<0.001	2.55	1.64–3.96	<0.001
ATRIA score	1.21	1.09–1.33	<0.001	1.35	1.18–1.54	<0.001
Duration of warfarin use	1.07	1.03–1.10	<0.001	1.07	1.03–1.11	<0.001
Peripheral embolism	2.77	1.04–7.38	0.04	4.82	1.26–18.46	0.02
HASBLED score	1.15	0.99–1.34	0.07	1.24	1.01–1.52	0.04
Active Ulcer	2.63	1.31–5.26	0.006			
HASBLED score ≥3	1.25	0.81–1.96	0.30			
Total warfarin dose/week	0.99	098–1.002	0.09			

*: Both minor and major bleedings were included in bleeding population. P<0.1 were entered into the multiple logistic regression analysis with forward stepwise method. ATRIA bleeding score: Anemia, severe renal disease, age ≥75 years, any prior hemorrhage, hypertension history; HASBLED: Hypertension history, abnormal liver and renal function, stroke history, bleeding history, labile INR, elderly, drugs; CI: Confidence interval; OR: Odds ratio.

### Predictors of Bleeding and Embolic Events

In the study group, minor (n=256, 40.9%) and major bleeding (n=90, 14.4%) events were noted in 346 (55.3%) patients during follow-up. Results of univariate and multivariate analyses for predicting bleeding events were presented in Table 3. In the whole study group, univariate analyses identified eight predictors of bleeding: Being a patient in VP group (p<0.001; OR 2.41 [1.66–3.50]), ATRIA score (p<0.001; OR 1.21 [1.09–1.33]), the duration of warfarin use (p<0.001; OR 1.07 [1.03–1.10]), peripheral embolism (p=0.04; OR 2.77 [1.04–7.38]), HASBLED score (p=0.07; OR 1.15 [0.99–1.34]), active ulcer (p=0.006; OR 2.63 [1.31–5.26]), HASBLED score ≥3 (p=0.30; OR 1.25 [0.81–1.96]), and total warfarin dose per week (p=0.09; OR 0.99 [0.98–1.002]). Multivariate analyses identified five independent predictors of bleeding: being a patient in the VP group (p<0.001; OR 2.55 [1.64–3.96]), ATRIA score (p<0.001; OR 1.35 [1.18–1.54]), the duration of warfarin use (p<0.001; OR 1.07 [1.03–1.11]), peripheral embolism (p=0.02; OR 4.82 [1.26–18.46]), and HASBLED score (p=0.04; OR 1.24 [1.01–1.52]).

In the whole study group, cerebral (n=69, 11.0%) and peripheral embolism (n=19, 3.0%) events were noted in 88 (14%) patients during follow-up. Results of univariate and multivariate analyses for predicting embolism were presented in Table 4. In the whole study group (n=625), univariate analyses identified four predictors of embolism complication occurrence: Being a patient in the VP group (p=0.001; OR 3.26 [1.59–6.66]), CHA_2_DS_2_-VASc score (p=0.001; OR 1.23 [1.09–1.39]), CHA_2_DS_2_-VASc score ≥2 (p=0.02; OR 1.88 [1.09–3.22]), and awareness of target INR value (p<0.001; OR 0.08 [1.08–4.50]). Multivariate analyses identified two independent predictors of embolism complication occurrence: Being a patient in the VP group (p=0.001; OR 3.42 [1.66–7.04]), CHA_2_DS_2_-VASc score (p<0.001; OR 1.28 [1.12–1.46]).

**Table 4.** Univariate and multivariate predictors of embolic events*

Variables	Univariate			Multivariate		
	OR	95% CI	p	OR	95% CI	p
CHA_2_DS_2_-VASc Score	1.23	1.09–1.39	0.001	1.28	1.12–1.46	<0.001
Variable physicians	3.26	1.59–6.66	0.001	3.42	1.66–7.04	0.001
CHA_2_DS_2_-VASc Score ≥2	1.88	1.09–3.22	0.02			
Awareness of target INR value	0.08	1.08–4.50	<0.001			

*: Both cerebral and peripheral embolisms were included in embolism population. P<0.1 were entered into the multiple logistic regression analysis with forward stepwise method. CHA_2_DS_2_-VASc: Congestive Heart Failure, Hypertension, age ≥75 years, diabetes mellitus, Prior stroke or TIA or thromboembolism, vascular disease, age 65–74 years, Sex, INR: International normalized ratio; CI: Confidence interval; OR: Odds ratio.

## Discussion

In this prospective cohort study, we demonstrated that warfarin monitoring with VP group was associated with lower TTR values and higher rates of cerebral embolism, minor and major bleeding events during follow-up. Although ATRIA and HASBLED bleeding scores were lower in VP group, there were more bleeding complications during follow-up. Moreover, although CHA_2_DS_2_-VASc Score was not different between two groups, embolic events were more frequent in VP group as compared to SP group during 3 years of follow-up. In multivariate analysis, warfarin monitoring by VP was found to be an independent predictor of both bleeding and thromboembolism.

The effect of gender on warfarin efficacy is still uncertain. In a previous study, it was revealed that male gender was associated with more effective TTR activity and TTR value [17, 18]. Although there were more males in the VP group in our study, we did not observe such a relationship between gender and complication rate, median TTR value and rate of effective TTR (TTR >65%). Another study evaluating the factors affecting INR variability revealed that the majority of the factors (52.8%) constituted from “unknown causes” and followed by patient noncompliance (19.8%), foods (13.2%), usage of concomitant drugs (10.0%), alcohol (3.1%), and herbal supplements (1.1%), respectively [19]. We postulate that “unknown causes” may potentially include variations of the physician monitoring of the patient, the doctor-patient relationship, and treatment compliance. A study investigating the patient compliance with anticoagulant treatment found that male gender, lack of a stable physician, and young age were the factors contributing to patient noncompliance [20]. The presence of a higher rate of male patients and a lower level of confidence about warfarin monitoring in the VP group may explain the lower TTR values in this group.

Patient-specific factors also influence TTR. Rose et al. found that younger age, female sex, lower income, black race, frequent hospitalizations, polypharmacy, active cancer, substance abuse, psychiatric disorders, dementia, and chronic liver disease were all independently associated with lower TTR [10, 21]. However, we believe that ineffectiveness of warfarin therapy cannot be explained solely with demographic and health characteristics of patients. The mechanisms underlying these effects are largely unknown, but non-adherence is likely contributing to some extent. The improvement in warfarin therapy and TTR is attributable to better clinic organization, dedicated and knowledgeable personnel. In this study, all participating centers were public (government) hospitals and had similar organizational process for the quality of the services and all of the patients were monitored by consultant cardiologists, and hence all were homogenous with regard to many aspects except having SP versus VP organization.

In several studies, compared to regular clinics, higher TTR (69% vs. 66%, p<0.001) was obtained from anticoagulation clinics where a licensed physician supervising advanced trained pharmacists and nurses [18, 22]. In another study from our country comparing the efficacy of monitoring clinics (anticoagulation vs. general cardiology clinic), it was found that the mean TTR was higher in anticoagulation clinic, furthermore total ischemic and major bleeding events were also higher in general cardiology outpatient clinics [23]. Likewise, in our study, mean TTR values were found to be higher in the SP group consisting stable consultant cardiologist similar to anticoagulation clinics. We believe that the exposure of patients to different physicians on different occasions have the potential to damage depth and accuracy of the relationship between physicians and patients, or alternatively, different physicians might have difficulty to assess and adapt to patient-specific conditions, which might affect INR targeting. Notably, this study differs from previous studies, which evaluated treatment quality in central anticoagulation clinics vs. public health services and also differs with the design that enable us to compare the treatment quality and outcome of patients on the basis of physician maintenance.

Although ATRIA and HASBLED bleeding scores of the patients in VP group were lower than the patients in SP group, the univariate and multivariate analyses designated that monitoring through “variable-different physicians” was an independent predictor of bleeding events. We consider that during monitoring by a SP, the physician relies on a better knowledge about the patient’s status and a higher level of confidence between the patient and physician, and treatment compliance, and thus performs better dose adjustment. Besides, VP group ordered more INR measurements compared to SP group, which could increase the healthcare costs.

A previous study showed that higher socioeconomic status was associated with higher TTR values [24]. It was also shown that a higher socioeconomic status and education increased patient’s compliance to treatment [25]. Lower TTR values and higher event rates were recorded in VP group, although socioeconomic status was higher in this group. The potential benefit of higher socioeconomic status was thought to be balanced by the monitoring by variable physician. In our study, although socioeconomic status was higher in VP group, the ratio of moderate-to-high level of confidence about warfarin monitoring was lower in this group. These findings potentially designate that patients’ compliance and confidence might be more important than socioeconomic status for effective treatment and outcomes. The contact of patients with a same-stable physician seems to improve the patient-physician relationship and thus, the patient’s level of compliance seems higher in these centers. As a result, contacting the same physician at each visit seems to increase the level of confidence about the treatment. A similar recent study investigating the factors affecting the patients’ compliance with INR monitoring showed that assigning an anticoagulation service provider constantly caring for the same patient increased the patient’s compliance with the treatment [26]. We postulate that this finding supports the efficacy of monitoring by the same and stable physician both for treatment success and patient compliance. With these findings, it can be speculated that this group of patients who were monitored by SP more likely to adopt patient self-testing of warfarin with remote control.

The results of our study should be considered within the context of its limitations. This is a multicenter study including 625 patients with a relatively small sample size. However, its results including mean TTR values were comparable with previous randomized studies. Second, there is a marked difference in the number of patients included between groups but similar findings were found on the basis of the clinical variables of the patients. Third, though, this study has a prospective cohort design, it only makes a comparison of two different settings of managing warfarin treatment, not comparing the different healthcare centers. This study solely aimed to investigate the “physician maintenance” on the influence of patient monitoring and compliance. Hence, it is difficult to generalize the results of this study to different health system profiles. Concerning, overall workload and practice patterns of cardiologists, the differences observed in the present study cannot be generalized to all physicians, particularly those with relatively low workload contrary to the centers involved in this study, which might enable better patient-physician relationship. We found a significant difference in the monitoring of patients taking warfarin, but more research is needed in this area.

### Conclusion

This study concludes that monitoring by the same-stable physicians improves the quality of care and hence influences the outcome of patients on warfarin. The net clinical benefit of this effect was observed through a decrease in bleeding and embolism. Clinicians should be encouraged to assess patient’s knowledge, confidence and awareness of his/her disease, patients’ present mood status, educational, socioeconomic status, and their effect on patients’ INR quality. “Same-stable-physician approach” seems to be effective, inexpensive and safe for anticoagulant management and it can be adopted as a feasible alternative model especially for patients having a very high level of risk of complications.
